# Editorial: Modern cultivation techniques for medicinal plants: impact on yield and secondary metabolite production

**DOI:** 10.3389/fpls.2026.1823253

**Published:** 2026-03-24

**Authors:** Sanja Radman, Silvana Nicola, Nina Kacjan-Marsic, Saeid Hazrati

**Affiliations:** 1 Department of Vegetable Crops, Faculty of Agriculture, University of Zagreb, Zagreb, Croatia; 2 Department of Agricultural, Forest and Food Sciences, University of Turin, Turin, Italy; 3 Agronomy Department, Biotechnical Faculty, University of Ljubljana, Ljubljana, Slovenia; 4 Department of Agronomy and Plant Breeding, Faculty of Agriculture, Azarbaijan Shahid Madani University, Tabriz, Iran

**Keywords:** climate-resilient cultivation, controlled environment agriculture (CEA), integrated fertilization approach, medicinal plants, secondary metabolites, sustainable cultivation practices

Medicinal plants are a vital source of bioactive secondary metabolites, including terpenoids, alkaloids, phenylpropanoids, and saponins, which have pharmaceutical, nutraceutical, and therapeutic applications (El-Saadony et al., 2025). However, as climate change, resource scarcity and unsustainable harvesting practices accelerate habitat destruction (Latif and Nawaz, 2025), the cultivation of medicinal plants must transition from traditional methods to more resilient, resource-efficient and technologically advanced production systems. The phytochemical profiles of cultivated medicinal plants are strongly influenced by factors such as growing systems, fertilization regimes, irrigation management, light conditions, and biostimulant applications (Zhang et al.).

**Figure 1:**
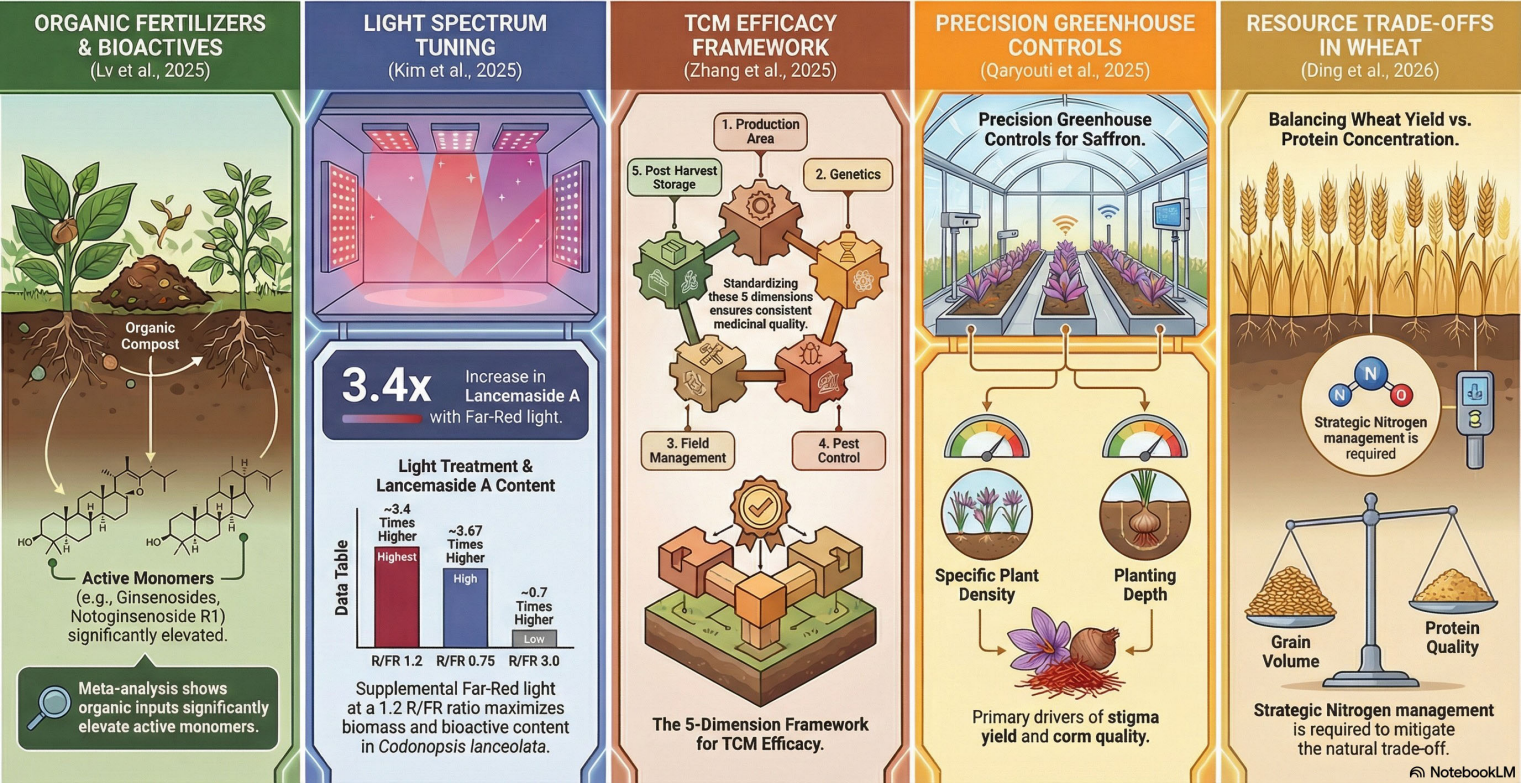
Graphic representation of the five articles presented in the Research Topic (Infographic generated with NotebookLM, developed by Google LLC, 1600 Amphitheatre Parkway, Mountain View, California, USA).

This Research Topic, entitled ‘Modern Cultivation Techniques for Medicinal Plants: Impact on Yield and Secondary Metabolite Production’, examines recent advances in the cultivation of medicinal and aromatic plants with the aim of maintaining or enhancing product quality and yield. The five articles in this Research Topic address topics such as controlled vertical farming and LED lighting, greenhouse-based saffron cultivation, quality-determining factors across the cultivation cycle, rainfed agrosystems, and a meta-analytical assessment of the effects of fertilizers on secondary metabolite accumulation (see **Figure 1**).

In a meta-analysis of 966 outcomes from 29 studies, Lv et al. examined the impact of fertilizers on the levels of bioactive saponins in medicinal plants. Saponins are triterpenoid or steroidal glycosides with anti-inflammatory, immunomodulatory and antitumour activities (Moses et al., 2014) and responded differently to fertilization regimes. Inorganic fertilizers promoted saponins, including ginsenoside Rg1 in Panax ginseng and other compounds in *Paris polyphylla* Smith, *Dioscorea* spp. and *Platycodon grandiflorus* (Jacq.) A.DC. due to enhanced nutrient availability. However, long-term use of these fertilizers can lead to soil degradation and reduced crop quality. In contrast, organic fertilizers improved microbial activity and the rhizosphere, significantly increasing ginsenoside R1 and ginsenosides Rb2 and Re, albeit with slower nutrient release. The combined application of both types of fertilizer was the most effective, maximizing both the diversity and yield of Panax ginsenosides. These results suggest that integrated fertilization is an effective strategy for saponin-rich crops and has broader implications for the production of secondary metabolites.

As the Traditional Chinese Medicine industry shifts from wild harvesting to large-scale cultivation, maintaining consistent, therapeutically relevant levels of secondary metabolites remains a major challenge. In a systematic narrative review, Zhang et al. identified five interrelated quality determinants across the production cycle. Site selection is crucial, as light and temperature can cause 4–5-fold variations in active compounds within the same species, while genetic background fundamentally determines therapeutic potential. In field management, continuous monocropping disrupts the soil microbiome; excess nitrogen reduces total phenolics, whereas potassium enhances secondary metabolite accumulation. Non-standardized pesticide use leads to a 13.82% reduction in net ginsenoside content. Post-harvest practices are also decisive, with drying *Lavandula angustifolia* Mill. at 30°C producing 18% more essential oil than ambient drying. The authors recommend phenotype-assisted breeding, ecologically informed site zoning and the use of organic alternatives.

Saffron (*Crocus sativus* L.) poses cultivation challenges due to strict thermoperiodic requirements and exclusive corm propagation. In a greenhouse study in Saudi Arabia, Qaryouti et al. tested three planting densities (67, 100, and 200 corms m^-^²) and two depths (8 and 13 cm). An active-cooling system reduced internal temperatures by 15°C during critical stages, enabling flowering under arid conditions. High density (200 corms m^-^²) maximized stigma yield per area but reduced individual corm size, while low density (67 corms m^-^²) promoted larger, propagation-viable daughter corms. Deeper planting (13 cm) impaired regeneration, especially at high density. A moderate density (100 corms m^-^²) at 8 cm depth was recommended to balance yield and long-term propagation, supporting sustainable saffron production in extreme climates.

Ding et al. examined how cultivation practices, soil water retention, and nitrogen dynamics influence yield formation and quality traits in winter wheat under Loess Plateau conditions. Although the study focused on winter wheat, the findings regarding nitrogen dynamics, dry matter translocation and agronomic optimization provide valuable insights that can be applied to the cultivation of medicinal plants.

Kim et al. examined the effects of different red/far-red (R/FR) light ratios on growth and bioactive compound accumulation in *Codonopsis lanceolata* (Siebold & Zucc.) Benth sprouts cultivated within a vertical farming system. Plants were exposed to four light treatments under equal photosynthetic photon flux density, with far-red light supplementation used to modify the R/FR ratio. Far-red enrichment significantly promoted biomass accumulation, plant height, and leaf area, while also enhancing phenolic and flavonoid content as well as antioxidant capacity. The concentration of lancemaside A, a major bioactive triterpenoid saponin characteristic of *Codonopsis lanceolata* (Siebold & Zucc.) Benth, was higher under moderate far-red supplementation, with a slight decrease observed at the lowest R/FR ratio. These findings highlight the potential of optimized spectral management in controlled-environment agriculture to simultaneously enhance productivity and phytochemical quality.

Together, these studies demonstrate that the yield and phytochemical integrity of medicinal plants are dynamic outcomes of cultivation management rather than fixed traits. Integrated fertilization, multidimensional quality assurance, and controlled-environment technologies can enhance productivity while preserving secondary metabolite biosynthesis. Nevertheless, many biosynthetic and regulatory mechanisms underlying metabolite responses to agronomic practices remain unclear. Advancing high-throughput metabolomic and transcriptomic tools for non-model medicinal species can link cultivation strategies to molecular biosynthetic outcomes. As global demand surpasses sustainable wild supply, optimizing and standardizing cultivation practices is essential to ensure the ecological resilience and therapeutic reliability of medicinal plants.

Future research should integrate controlled environment agriculture, precision resource management, and systems-level molecular analyses to develop predictive cultivation models capable of stabilizing metabolite production under variable environmental conditions.

